# Targeted drug delivery via caveolae-associated protein PV1 improves lung fibrosis

**DOI:** 10.1038/s42003-019-0337-2

**Published:** 2019-03-07

**Authors:** Gabriela M. Marchetti, Timothy J. Burwell, Norman C. Peterson, Jennifer A. Cann, Richard N. Hanna, Qing Li, Emily L. Ongstad, Jonathan T. Boyd, Maureen A. Kennedy, Weiguang Zhao, Keith W. Rickert, Joseph S. Grimsby, William F. Dall’Acqua, Herren Wu, Ping Tsui, M. Jack Borrok, Ruchi Gupta

**Affiliations:** 1 Antibody Discovery & Protein Engineering, MedImmune, Gaithersburg, MD 20878 USA; 2 Cardiovascular & Metabolic Diseases, MedImmune, Gaithersburg, MD 20878 USA; 3 Respiratory, Inflammation and Autoimmune Diseases, MedImmune, Gaithersburg, MD 20878 USA; 4 Translational Sciences, MedImmune, Gaithersburg, MD 20878 USA; 5 Pathology, MedImmune, Gaithersburg, MD 20878 USA; 6 Microbial Sciences, MedImmune, Gaithersburg, MD 20878 USA

## Abstract

Systemic administration of bio-therapeutics can result in only a fraction of drug reaching targeted tissues, with the majority of drug being distributed to tissues irrelevant to the drug’s site of action. Targeted delivery to specific organs may allow for greater accumulation, better efficacy, and improved safety. We investigated how targeting plasmalemma vesicle-associated protein (PV1), a protein found in the endothelial caveolae of lungs and kidneys, can promote accumulation in these organs. Using ex vivo fluorescence imaging, we show that intravenously administered αPV1 antibodies localize to mouse lungs and kidneys. In a bleomycin-induced idiopathic pulmonary fibrosis (IPF) mouse model, αPV1 conjugated to Prostaglandin E_2_ (PGE_2_), a known anti-fibrotic agent, significantly reduced collagen content and fibrosis whereas a non-targeted PGE_2_ antibody conjugate failed to slow fibrosis progression. Our results demonstrate that PV1 targeting can be utilized to deliver therapeutics to lungs and this approach is potentially applicable for various lung diseases.

## Abstract (web summary)

Marchetti et al. show that antibodies against Plasmalemma Vesicle Associated Protein (PV1) can be used to efficiently target drugs to lungs and kidneys. In a bleomycin induced lung fibrosis model, mice treated with anti-PV1 antibodies linked to an anti-fibrotic drug exhibited reduced collagen deposition and fibrosis progression compared to control antibodies.

## Introduction

The use of monoclonal antibodies (mAbs) as therapeutics continues to expand for the treatment of cancer, inflammatory, autoimmune, cardiovascular, and infectious diseases. For proper efficacy, it is imperative for many therapeutic mAbs to distribute to and accumulate in a specific organ or tissue. Indeed, the local (tissue) concentration of an antibody, which is a fraction of the original dose, plays a key role in determining the efficacy of the therapeutic mAb. Targeted drug delivery to specific tissues has the potential to improve efficacy of both small and large molecule therapeutics, and prevent off-target toxicity. Such delivery mechanisms may be critical for targeting pulmonary diseases, such as fibrosis, chronic obstructive pulmonary disease (COPD), asthma, cancer, and microbial infections^1^.

Drug delivery into lungs is challenging due to its highly specialized vascular structure comprised of a continuous non-fenestrated endothelial monolayer. This endothelial barrier regulates the permeability of molecules from the blood and prevents entry into tissue compartments. Caveolae, 60–80 nm diameter invaginations present at high levels in lung endothelia, are an important gateway regulating transport of proteins across the endothelial layer^2^. In addition to protein transcytosis, caveolae functions in endocytosis, and calcium signaling^3,4^. Recently, tissue-specific delivery has been achieved using antibodies against specific caveolae proteins^5,6^. However, demonstrating that caveolae-mediated targeted delivery can improve therapeutic efficacy in a disease model has only been recently pursued^7^.

Plasmalemma vesicle-associated protein (PV1) is a known caveolae-associated protein^8^. In this study, we generated monoclonal and bispecific antibodies against PV1 and demonstrate targeting to lungs and kidneys upon systemic administration. We conjugated an anti-fibrotic small molecule to an anti-PV1 (αPV1) antibody and observe substantial reduction in the development of lung fibrosis (compared to an isotype control antibody) in an idiopathic pulmonary fibrosis (IPF) mouse model. This finding was confirmed using both immunohistochemistry (IHC) for alpha-1 type I collagen (Col1a1) and second harmonics imaging microscopy for fibrillar collagen. We demonstrate that a mouse-specific high-affinity αPV1 monoclonal antibody can be used to deliver disease-modulating therapeutics to lungs and kidneys of mice and show similar target expression of PV1 in human normal and diseased lungs.

## Results

### PV1 expression in mouse

**Fig. 1:**
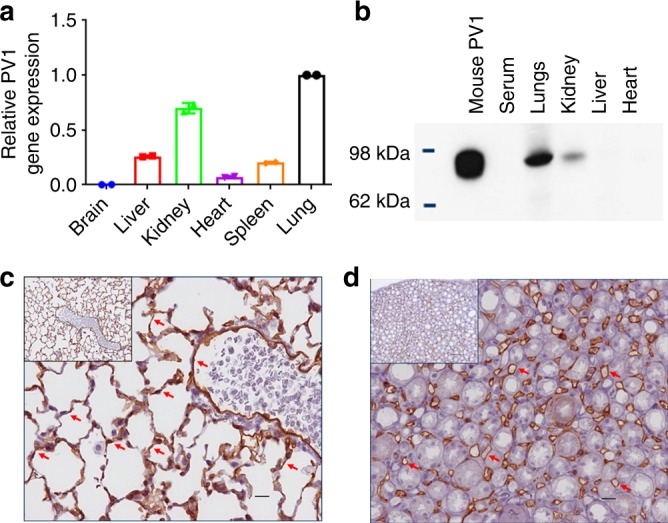
Plasmalemma vesicle-associated protein (PV1) expression. Relative mRNA of PV1 compared with β-actin mRNA in different tissues, highest expression in lungs followed by kidneys (**a**). Western blotting of mouse tissue homogenate (25 ng of recombinant mouse PV1 and 50 μg total protein from tissue lysates). PV1 dimer was detected in lungs and kidneys (**b**). αPV1 immunohistochemistry of normal mouse lung (**c**) and kidney (**d**) showed PV1 expression in endothelial cells lining large vessels and small capillaries throughout the lung (alveolar capillaries) and the renal medulla (vasa recta). Scale bars: 10 μm

A rat-specific αPV1 antibody had previously been shown to localize to lungs and kidneys in rats^9^. To determine whether similar antibody homing could be achieved in mice, tissues were assessed for PV1 expression via qPCR for mRNA levels and through western blot for protein expression. At the mRNA level, the most prominent PV1 expression was observed in lungs, followed by kidneys and liver (Fig. 1a). At the protein expression level, western blots of whole tissue homogenates identified dimeric PV1 in lungs and kidneys (Fig. 1b). IHC further confirmed PV1 expression in lungs and kidneys. IHC showed strong expression of PV1 in endothelial cells lining all vessels throughout the lung, including medium and large caliber arteries and veins, as well as alveolar capillaries (Fig. 1c). In the kidney, a similar pattern was seen with strong expression in endothelial cells lining arteries, veins, peritubular capillaries, and vasa recta; however, capillaries within the glomerular tufts were consistently negative (Fig. 1d).

In addition to healthy lungs, we assessed PV1 expression in bleomycin-treated fibrotic mouse lungs. Western blots of tissue homogenates taken from mice that had been administered bleomycin for 28 days via an osmotic pump showed PV1 protein expression profiles similar to that of untreated mice (Supplementary Figure 1). PV1 expression was detected in the kidneys and lungs of both untreated and bleomycin-treated mice but was not apparent in spleen, heart, or liver.

### Generation of the antibody against mouse PV1

Meca32 is a high-affinity rat anti-mouse antibody targeting PV1  commonly used for IHC. We determined the sequence of Meca32 via de novo protein sequencing^10^. Identification of Meca32 protein sequence allowed for generation of novel constructs including bispecific antibodies. Several variants, differing in isobaric isoleucine/leucine residues in the variable light (VL) and variable heavy (VH) complementarity-determining regions (CDRs) were generated as Fabs and binding was compared to commercial Meca32 Fab. All tested variants bound similarly to commercial Meca32. One variant (hereafter referred to as αPV1 IgG) was chosen to be expressed as both a human IgG1 chimeric antibody and as an ScFv in the bispecific (Bis3) format^11^. The Bis3 bispecific was generated with an anti-*Pseudomonas* sp. antibody Cam003 as the IgG arm and the αPV1 variant as an scFv C-terminal heavy chain fusion^11,12^. The BiS3 format was chosen to allow for maximum distance between the separate binding arms with minimum interference. Sequence information for the VH and VL domains are shown in Supplementary Figure 2A. In a Fab ELISA, the de novo sequenced αPV1 had very similar EC_50_ values to commercial Meca32 Fab (Supplementary Figure 2B). In the IgG1 and Bis3 format, the antibodies retained similar binding to recombinant mouse PV1 (Supplementary Figure 1C). Additional characterization of both antibody formats via surface plasmon resonance (SPR) revealed *K*_D_ values of 0.5 nM for the IgG and 0.2 nM for the Bis3 bispecific antibody (Supplementary Figure 2D).

### Tissue homing of αPV1 antibody after intravenous (IV) injection

**Fig. 2:**
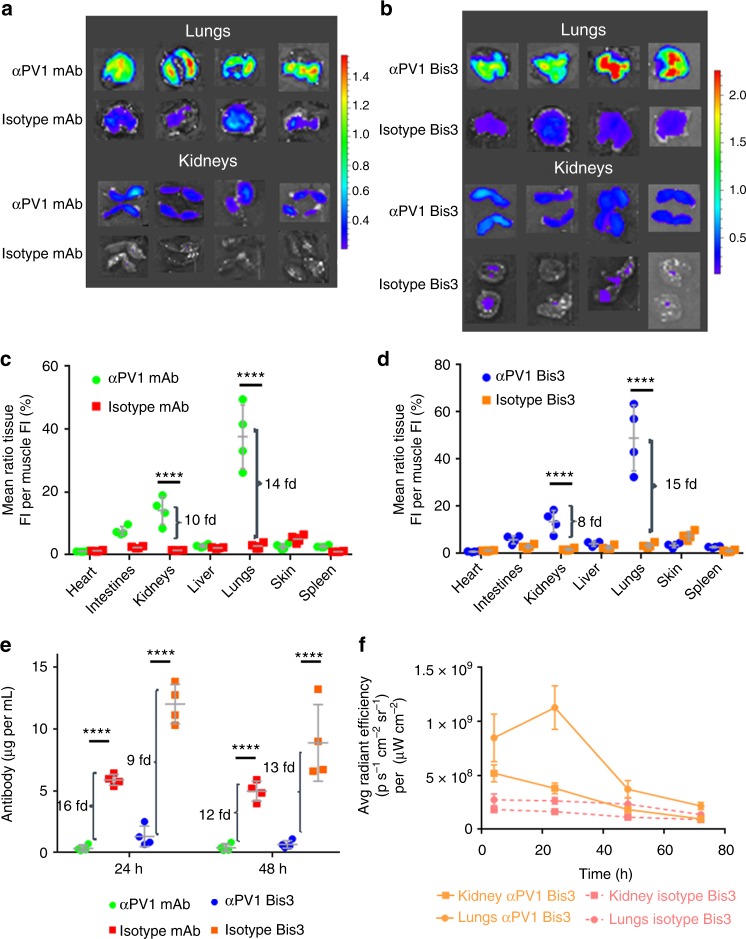
Localization of αPV1 constructs to lung and kidney via ex vivo imaging. Ex vivo imaging of lung and kidney shows higher fluorescence intensity in the lungs and kidneys of mice injected with 2 mg/kg of Alexa Fluor 680-labeled αPV1 monoclonal (**a**) and bispecific (**b**) antibodies compared with respective Alexa Fluor 680-labeled isotype controls at 24 h after intravenous injection. Quantification of fluorescence intensity from ex vivo imaging was normalized to the skeletal muscle (mean + SD) for αPV1 vs. isotype mAb (**c**) and for the bispecific αPV1 construct and bispecific isotype (**d**). Brackets indicate fold change in the fluorescence intensity of αPV1 to isotype control in lungs and kidneys. Serum antibody concentrations from the mice were quantified at 24 and 48 h (**e**). Tissue homing of the αPV1 results in rapid clearance of the antibody from the blood. Brackets indicate fold change in serum concentration of isotype to αPV1 antibodies. A time-course of ex vivo imaging for the bispecific αPV1 construct and bispecific isotype (**f**) shows lung accumulation peaking at 24 h post dosage where isotype levels are lower and relatively stable. Data are shown as the mean ± SD; ****p < 0.0001, *n* = 4 per group

To assess the biodistribution of αPV1 antibodies, fluorophore-labeled αPV1 antibody and isotype control antibody were injected IV into Balb/c mice at 2 mg per kg. Ex vivo surface fluorescence (normalized to skeletal muscle) in the lungs and kidneys of mice injected with αPV1 monoclonal Ab was markedly higher than those of mice injected with isotype control (Fig. 2a). As the newly constructed αPV1 antibody exhibited highly increased lung and kidney localization, we sought to determine whether this construct would retain targeting in the bispecific format to home an additional antigen-binding arm to these organs. Similar lung and kidney localization was observed with the αPV1 Bis3 molecule when compared to its respective Bis3 isotype control (Fig. 2b). Quantification of ex vivo imaging revealed αPV1 IgG had 14-fold more normalized fluorescence signal in lungs than the isotype control and 10-fold more fluorescence signal in the kidneys (Fig. 2c). The bispecific αPV1 construct homed to lungs and kidneys in a similar manner with ex vivo surface fluorescence 15-fold higher than isotype and kidneys having eight-fold more signal than the isotype Bis3 (Fig. 2d). In addition to healthy mice, αPV1 antibody accumulation was assessed in bleomycin-treated fibrotic lung as well kidneys (Supplementary Figure 3). Significant increase was observed in αPV1 Bis3 levels in both untreated mice and in mice with bleomycin-induced pulmonary fibrosis (28 days of bleomycin infusion through osmotic pump) compared to isotype Bis3 levels. No statistical differences in lung fluorescence signal were observed between αPV1 Bis3 in untreated and bleomycin-treated mice.

We next evaluated the level of both monospecific and bispecific αPV1 antibodies in mouse serum. The amount of αPV1 IgG and Bis3 in the blood were markedly lower than the isotype control antibodies (Fig. 2e). At 24 h after injection, serum had 16-fold less αPV1 IgG and αPV1 Bis3 was 9-fold less when compared with the Isotype control. At 48 h after injection, serum levels were reduced by ~12-fold for both αPV1 IgG and Bis3 in comparison to the representative isotype controls.

An additional ex vivo fluorescence imaging experiment was performed to assess lung and kidney accumulation at multiple timepoints (Fig. 2f). Mice lungs and kidneys were analyzed at 4, 24, 48, and 72 h after injection of labeled αPV1 Bis3 and isotype Bis3. Lung fluorescence signal peaked at 24 h, and then gradually declined to near isotype levels. Kidney signal peaked at 4 h and then decreased to near isotype levels within 48 h.

### Localization of αPV1 antibody in lung and kidney endothelium

**Fig. 3:**
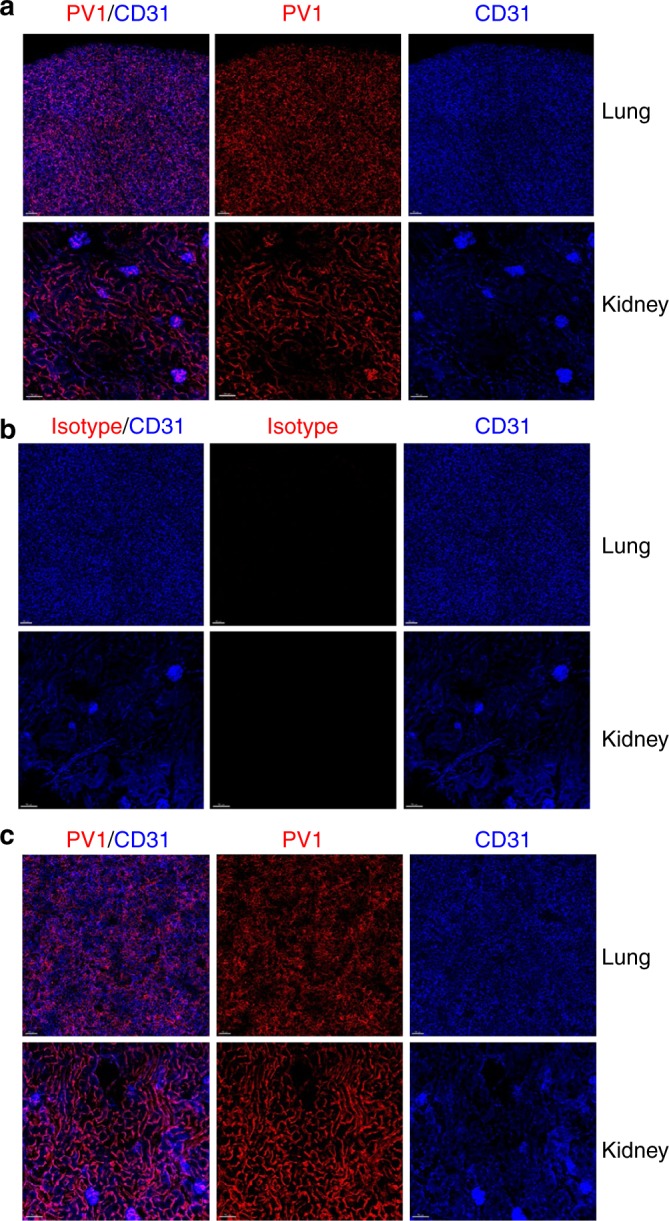
Lung and kidney homing of αPV1 antibody captured by confocal microscopy. Mouse lung and kidneys were analyzed by confocal microscopy 4 h after IV administration with either Alexa-594 αPV1 BiS3 (**a**, **c**) or Alexa-594 isotype Bis3 (**b**). Intense PV1 staining (red) can be observed in both untreated (**a**) and in bleomycin-treated mouse lung and kidney (**c**). The PV1 staining pattern is similar to the CD31-BV421 staining (blue) pattern revealing a mostly endothelial distribution in the lungs and kidneys. Little to no specific staining in lungs or kidney was observed with an isotype control antibody (**b**). Images were acquired using Zeiss 880 Airyscan microscope. Bars = 70 µm

We next used confocal microscopy to further examine the lung and kidney tissue homing of αPV1 antibodies in untreated and bleomycin-treated mice. Alexa 594-labeled αPV1 Bis3 and isotype Bis3 antibodies were injected into mouse tail veins. At 24 h, samples of tissues (lungs and kidneys) were then processed for confocal imaging to determine localization of the constructs within the specific organs. A labeled anti-mouse CD31 antibody was intravenously injected 15 min prior to dissection to provide contrast and stain the endothelia. In untreated mice, the antibody targeting PV1 localized to endothelial cells lining the lung endothelia, with substantial co-staining with the labeled anti-CD31 antibody (Fig. 3a). In the kidneys, staining was again similar to that of the epithelial marker CD31 with substantial accumulation in the tubules and weaker signal in the glomeruli. At 24 h, no staining in kidney or lung was observed for the labeled Bis3 isotype (Fig. 3b). Staining in bleomycin-treated mice with fibrotic lungs (Fig. 3c) was consistent with that of untreated mice (Fig. 3a).

### αPV1-PGE_2_ conjugate improves lung fibrosis in mice

After observing that αPV1 antibodies homed to both healthy and fibrotic lungs, we next sought to determine whether this tissue targeting could be utilized to shuttle drugs to fibrotic lungs. Prostaglandin E_2_ (PGE_2_) is a short-lived eicosanoid hormone with known anti-fibrotic activity^13,14^. Due to its short half-life and pleotropic effects in other tissues, we hypothesized that conjugation to a PV1-targeting antibody, could localize its antifibrotic effect to the lungs while avoiding systemic release of the drug. To this end, αPV1 and isotype control antibodies were conjugated to PGE_2_. Each construct was conjugated with ~10 μg of PGE_2_ per mg of antibody in the samples. Both αPV1 and isotype-conjugated constructs showed similar PGE_2_ activity (Supplementary Figure 4A). αPV1-PGE_2_ retained binding to recombinant mouse PV1 with slightly reduced IC_50_ values compared to the unconjugated PV1 antibody (0.46 µg/mL compared to 0.11 µg/mL, respectively) (Supplementary Figure 4B).

**Fig. 4:**
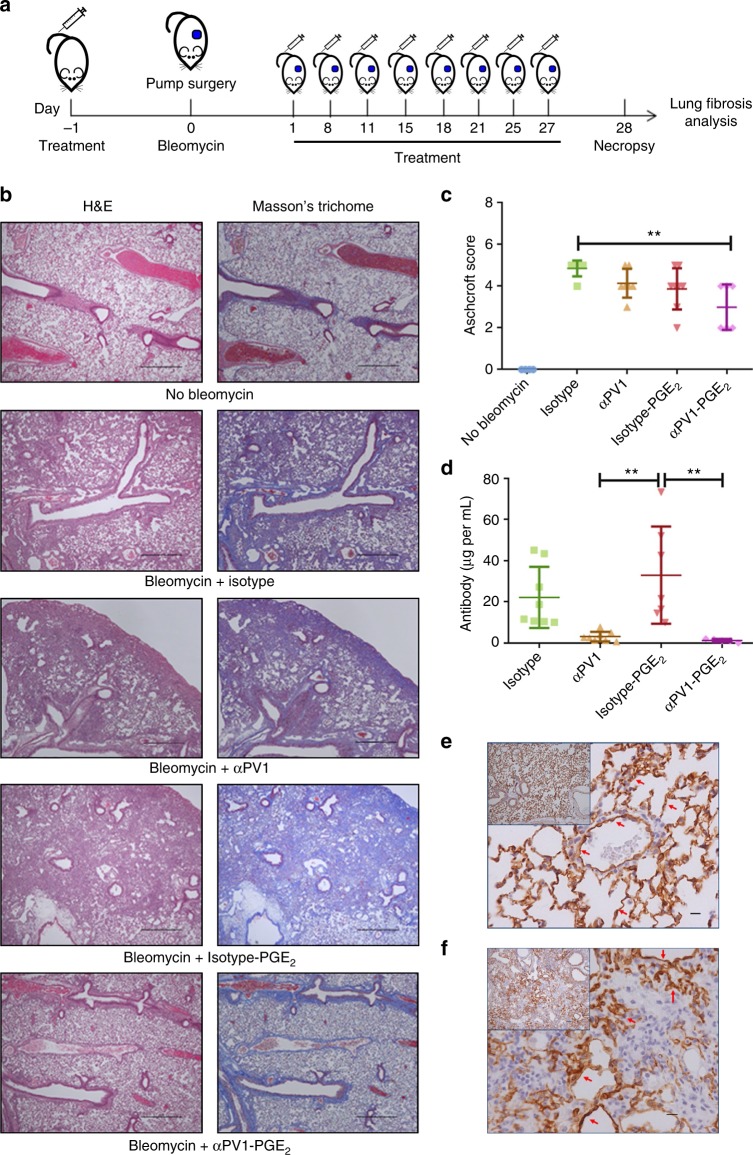
Anti-fibrotic activity of αPV1-PGE_2_ in bleomycin-induced lung fibrosis. Schematic view of administration protocol (**a**). Representative images of lungs from mice from each treatment group are shown stained with H&E, and Masson’s Trichrome to highlight fibrosis (blue). The abundant extracellular matrix (ECM) deposition in the lungs from bleomycin isotype groups was substantially reduced in the lungs of animals treated with αPV1-PGE_2_; scale bars: 100 μm. Lung histological alterations were scored using a modified Ashcroft scale system. Reduction in the levels of lung fibrosis was observed for αPV1-PGE_2_-treated animals (**c**). Rapid reduction in serum antibody levels for the αPV1 groups was observed which is consistent with fast lung and kidney accumulation (**d**). αPV1 immunohistochemistry of lungs from control mice (**e**) and mice receiving bleomycin (**f**) illustrate PV1 expression remains high even in fibrotic lung tissue. Data represent mean ± SD. One-way ANOVA (Tukey post-hoc analyses) was used to evaluate the statistical significance (defined as a *p-*value < 0.05). *p < 0.05; **p < 0.01; ***p < 0.001; scale bars = 10 μm; *n* = 8

Isotype-PGE_2_ and αPV1-PGE_2_ as well as unconjugated αPV1 and isotype antibodies were dosed as per the schematic shown in Fig. 4a. Analysis of mouse lungs by IHC revealed the deposition of abundant extracellular matrix (ECM) proteins and fibroblasts in the lungs of bleomycin administered mice treated with isotype. However, ECM was substantially reduced in the lungs of αPV1-PGE_2_-treated mice (Fig. 4b). The Ashcroft score, used for quantification of total fibrosis, was significantly reduced in the αPV1-PGE_2_-treated group when compared to the Isotype group (*p* < 0.001) (Fig. 4c). Conversely, treatment with isotype conjugated to PGE_2_ did not impact the degree of fibrosis in the lung compared to isotype or αPV1 groups.

Treatment with αPV1-PGE_2_ also reduced mRNA transcript expression of fibrotic markers including Fibroblast Grown Factor 2 (FGF2), Collagen I and III (Col1A1 and Col3A1) and Fibronectin (Fn1) (Supplementary Figure 5A–D). Importantly, profibrotic mediator TGF-β and TGFβ-receptors (TGFBR1 and TGFBR2) were significantly reduced in mice treated with αPV1-PGE_2_ (one-way ANOVA, *p* < 0.05; Supplementary Figure 5E–H). Conversely, the isotype-PGE_2_ did not significantly reduce fibrotic marker transcript levels when compared to isotype-treated groups.

Serum quantification of the antibodies on day 28 showed that isotype antibodies remained in the serum after this administration regimen while levels of PV1-targeted antibodies were significantly reduced in the sera (one-way ANOVA, *p* < 0.01; Fig. 4d). The decreased serum levels of the αPV1 antibodies are consistent with rapid tissue uptake and serum depletion observed in untreated mice (Fig. 2e). The rapid clearance from blood due to accumulation in the target tissue was also seen in the αPV1-PGE_2_ group suggesting an effective delivery of PGE_2_.

Mice from untreated and bleomycin-treated groups were also evaluated for expression of PV1 via IHC (Fig. 4e, f). Extensive PV1 expression was observed in the mouse endothelium for both untreated mouse lungs, as well as for bleomycin-treated fibrotic lungs (Fig. 4f).

### Reduction in collagen levels in αPV1-PGE_2_-treated mice lungs

**Fig. 5:**
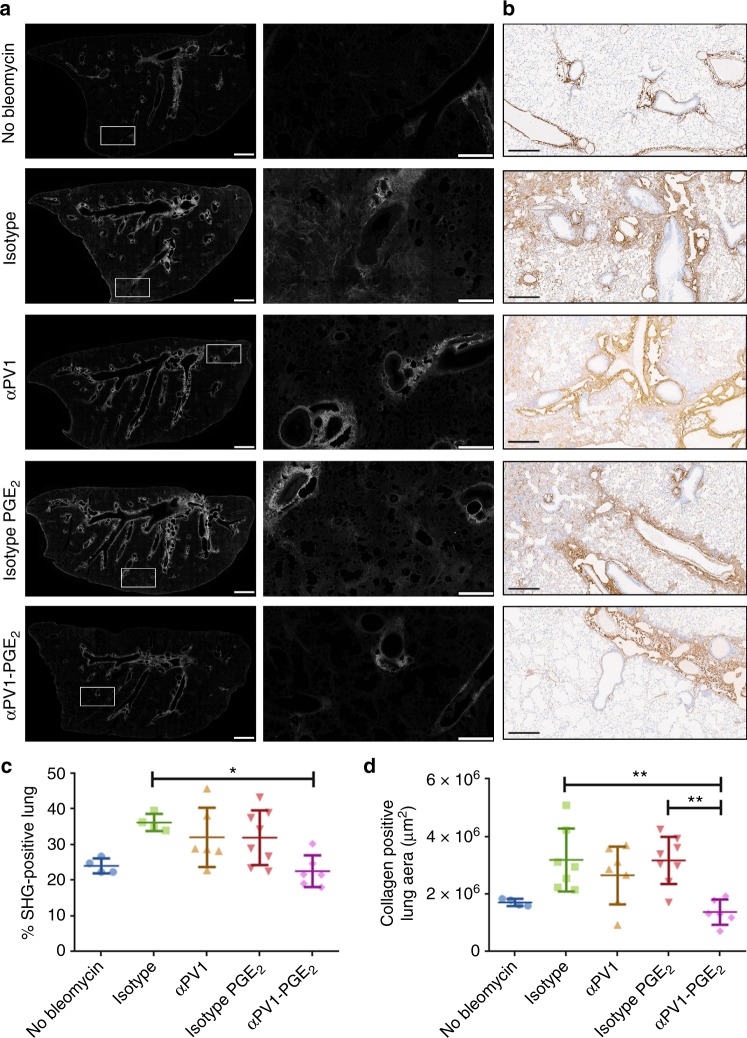
Reduced collagen deposition in bleomycin-induced fibrotic lungs treated with αPV1-PGE_2_. Representative second harmonic generation (SHG) images highlighting fibrillar collagen (white) deposition in mouse lung slices are shown (**a**). Zoomed-in images of insets (white rectangles) are shown in the middle columns. Scale bars are 1 mm for images in the first column and 250 μm for the images in the middle columns. Representative IHC images are shown generated using an anti-Cola1a antibody (**b**); scale bars: 300 μm. Quantitative analysis of SHG images reveal decreased fibrillar collagen in the αPV1-PGE_2_-treated group (**c**). Percent positive collagen a1a area in collagen a1a lungs are also quantified again revealing decreased collagen deposition in αPV1-PGE_2_-treated animals (**d**). Data represent mean ± SD. One-way ANOVA (Tukey post-hoc analyses) was used to evaluate the statistical significance. *p < 0.05; **p < 0.01; scale bars = 10 μm; *n* = 8

Having shown a reduction in overall fibrosis in the lung through αPV1 delivery of PGE_2_, we next sought to specifically determine whether PGE_2_-conjugated αPV1 antibodies influenced collagen content in the lung. To accomplish this, we quantified collagen in the lung using second harmonic generation (SHG), a label-free, highly specific and highly sensitive technique for detecting fibrillar collagen (Fig. 5a)^15,16^. Tile scan images of the entire lung cross-section demonstrated low percent SHG-positive lung in animals treated with αPV1-PGE_2_ compared to isotype control or isotype-PGE_2_ (Fig. 5a). Collagen 1a1 immunohistochemical staining demonstrated similar patterns and extent of fibrosis as those detected by SHG (Fig. 5b). Quantitative analysis of both SHG lung and Collagen 1a1 IHC (Fig. 5c, d) consistently showed reduced collagen content in the αPV1-PGE_2_-treated group compared to other bleomycin administered groups.

### PV1 expression in human tissues

**Fig. 6:**
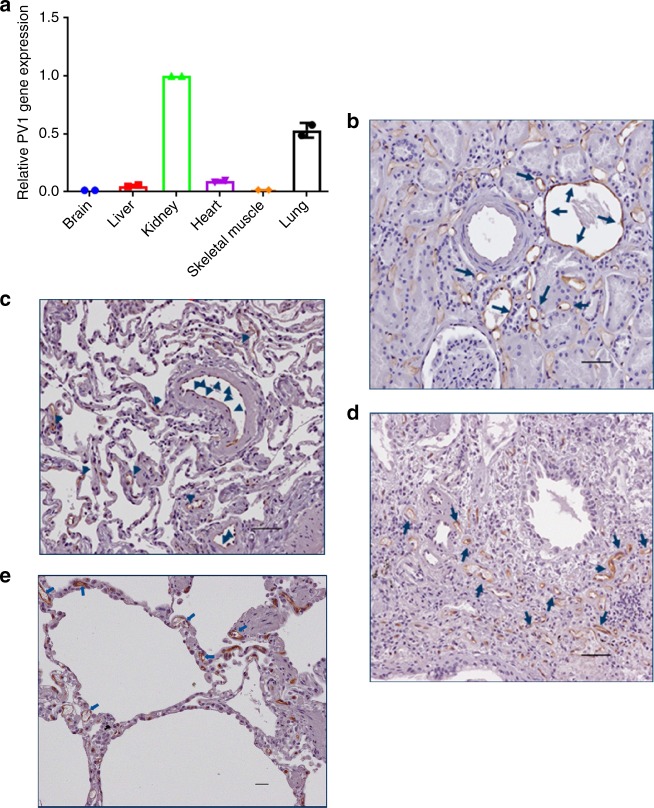
Plasmalemma vesicle-associated protein (PV1) expression in humans. (**a**) Relative mRNA of PV1 compared with GAPDH mRNA in different tissues is shown. αPV1 immunohistochemistry of normal human kidney (**b**) and lung (**c**), and human IPF (**d**) and COPD (**e**) lung, showing PV1 expression in endothelial cells lining large vessels and small capillaries, including the alveolar capillaries in the lung, and the peritubular capillaries in the renal cortex. Images are representative of tissue sections from at least three donors each. Scale bars = 10 µm

To assess the translatability of PV1-mediated lung and kidney targeting in humans, PV1 expression was analyzed in normal and diseased human tissues. Gene expression profile of PV1 in normal human tissues shows high expression in kidneys and lungs (Fig. 6a), suggesting that PV1-targeting strategies may be translatable to humans. The protein expression pattern of PV1 in normal human lung and kidney was examined by IHC (Fig. 6b, c), as well as human lung samples from IPF and COPD patients (Fig. 6d, e). Analogous to the expression observed in mouse, normal human lung and kidney revealed endothelial cell expression of PV1 in all vessel types (arteries, veins, capillaries) except glomerular capillaries. In COPD and IPF diseased lungs, strong endothelial cell expression was also observed.

## Discussion

Endothelial cells compose a unique semi-permeable barrier between the vascular lumen and surrounding tissue. The concept of targeting proteins on the endothelial cell surface to facilitate tissue and organ-specific drug delivery is well established^17^. Nevertheless, the lack of viable, safe, clinically translatable targets, as well as a dearth of studies demonstrating improved efficacy for targeted drugs have hindered progress in the field. For example, antibodies targeting either angiotensin-converting enzyme (ACE) or thrombomodulin accumulate in the lungs at high levels due to target enrichment^13^. However, interventions with ACE-targeting antibodies have led to hypertension, cough, and edema while targeting thrombomodulin can trigger thrombosis^14^. Anti-ICAM-1 and anti-PECAM antibodies also accumulate in the lungs after IV delivery. Unlike ACE-targeting antibodies, both anti-ICAM-1 and anti-PECAM antibodies do not internalize and remain on the cell surface. Nevertheless, multivalent anti-PECAM conjugates showed a five-fold increase in lung homing and internalization via endocytosis^18^.

In this study, the caveolae structural protein, PV1 was assessed for feasibility as a tissue delivery target for mAbs. It has previously been shown that antibodies targeting rat PV1 accumulate in rat lungs and kidneys^9,19^. Additionally, antibodies targeting PV1 have been shown to accumulate in mouse lungs^20^. Curiously, this same study demonstrated that nanoparticles targeting PV1 did not accumulate in mouse lungs, possibly indicating a limitation for PV1 localization. In mice, PV1 is predominantly expressed in both lungs and kidneys (Fig. 1). The expression was localized to both micro and macro vessels of the lung and kidney vasculature. Using ex vivo fluorescent imaging, we demonstrated that αPV1 antibodies homed to lungs and kidneys, in contrast to labeled isotype antibodies (Fig. 2). Confocal imaging of the mouse lung and the kidney tissues after systemic administration of fluorescently labeled antibodies revealed prominent endothelial localization in both tissues (Fig. 3). A bispecific antibody targeting PV1 showed similar level of accumulation in lungs and kidneys over isotype bispecific Ab. Both the IgG and Bis3 αPV1 antibodies accumulated in lungs and kidneys at similar levels and rates despite the small affinity differences between them. Notably, a substantial reduction in the serum concentration of both monoclonal and bispecific PV1-targeted antibodies was observed concurrent with tissue accumulation.

Building on these results, we assessed whether PV1 lung endothelial targeting could improve the efficacy of the anti-fibrotic small molecule, PGE_2_, in a bleomycin-induced pulmonary fibrosis model. Bleomycin-induced pulmonary fibrosis in rodents has been widely used as a means of studying the mechanisms involved in fibrosis and the evaluation of potential therapies for IPF. The systemic delivery of bleomycin via osmotic pumps mimics pleural fibrosis observed in human pulmonary fibrosis^21,22^. In this model, we demonstrate that the αPV1 mAb localizes to the fibrotic lung in an identical manner as in the normal lung. Although anti-fibrotic activity for PGE_2_ alone has been demonstrated previously (by continuous osmotic pump delivery) in the bleomycin-induced IPF model^23^, the short half-life (~5 min in serum) and known abortifacient activity^24^ hinder the development of the molecule as an anti-fibrotic therapeutic in humans. To enhance the delivery of the PGE_2_ to lung tissue, we conjugated PGE_2_ to either αPV1 targeting or isotype antibodies, and administered the drug over 4 weeks concurrent with continuous bleomycin exposure. After the study, a substantial reduction in collagen deposition in lungs of mice treated with αPV1 conjugated to PGE_2_ in comparison to mice treated with isotype-PGE_2_ was observed along with decreased mRNA levels of several transcripts associated with ECM remodeling and fibrosis. To further quantify the change in collagen content in the lung we utilized the innovative technique of SHG to show the decrease in fibrillar collagen content with αPV1-PGE_2_ treatment. The results were further confirmed with quantitative collagen1a1 IHC. Additionally, we demonstrated that the PV1 protein expression was present in normal human lung, as well as in IPF and COPD diseased lungs (Fig. 6). This similar expression profiles across species suggest PV1-mediated lung homing may also be possible in humans. Further studies with non-human primates will be needed to fully assess whether the PV1 targeting seen in rodents could be recapitulated in primates.

By targeting the caveolae-associated protein PV1, we have shown that we can greatly increase the local concentration of both a bispecific antibody and a small molecule conjugated antibody in both lungs and kidneys. We demonstrated the PV1-targeted localization in both normal and diseased tissue. Serum levels of the PV1-targeting antibodies substantially decreased concurrent with the tissue targeting as has been observed previously with another caveolae-associated protein aminopeptidase P2 (APP2) in rats^5^. This robust partitioning observed with certain caveolae-associated proteins may be a beneficial attribute for reducing off target drug-toxicity and increasing on target drug concentration and in turn efficacy. It may also allow for administration of lower doses which is critical for diseases like COPD which can require drug combinations. Additionally, the unique ability to target lung and kidney simultaneously through PV1 may allow for the treatment of comorbidities of the lungs and kidneys. Overall, targeted organ delivery via PV1 has the potential to improve drug efficacy as well as safety.

## Methods

### Ethics statement

All animal studies were approved by the MedImmune IACUC and were conducted in an Association for Assessment and Accreditation of Laboratory Animal Care–accredited facility in compliance with US regulations governing the housing and use of animals.

### Recombinant antibody production

All constructs were expressed transiently in HEK293F cells using 293fectin™ (Invitrogen) and grown in Invitrogen’s serum-free Freestyle™ medium. The culture medium was collected 10 days after transfection, and antibodies were purified by standard protein A affinity chromatography in accordance with the manufacturer’s protocol (GE Healthcare, Piscataway, NJ). If necessary, antibody constructs were then fractionated via size-exclusion chromatography to attain > 95% monomeric content.

### Imaging study

Anti-PV1 antibody and an isotype control antibody were labeled with Alexa fluor 680 (SAIVI Rapid Antibody Labeling Kit, Life Technologies, Grand Island, NY—degree of labeling (DOL) = 2–3) and injected IV into Balb/c mice at 2 mg/kg. Both antibodies were labeled with the same DOL and injected into separate groups of mice at the same dose (2 mg/kg). Blood and major tissues were harvested from euthanized mice at 24 and 48 h after the injection of the antibodies. The surface fluorescence from anesthetized mice (2% isoflurane) and tissues (ex vivo) was analyzed by using an IVIS Spectrum (Perkin Elmer) set at medium binning, F-stop 1, and auto exposure. Fluorescent signals were standardized across mice and presented as a proportion of the fluorescence from the skeletal muscle (ex vivo)^25^. After this standardization across mice and skeletal muscle the tissue accumulation data was done dividing targeted (αPV1) by non-targeted (isotype ab) groups. Data were analyzed with GraphPad 6.03 software (Prism, La Jolla, CA). ANOVA; Tukey post hoc analyses were preformed to determine statistical significance (defined as a *p*-value < 0.05).

### Second harmonics generation imaging

Formalin-fixed paraffin-embedded (FFPE) lung tissues were sectioned at 20 µm, mounted, and imaged unstained for SHG signals. Samples were imaged on a Leica SP8 Dive equipped with a multi-photon Insight X3 Laser (Spectra Physics, Santa Clara, CA) providing two-photon excitation at 920 nm, subjected to 10 nM bandpass around the excitation wavelength and signal collected with a HyD detector. Samples were imaged with a ×40 1.3NA PlanApo objective. Laser power and gain were kept constant across all samples over ~12 h tile scan acquisition time. If multi-photon laser power varied, images were excluded from analysis. The entire tissue section was imaged by obtaining a tile scan of each section with z-stacks (1 µm sections) over the entire tissue thickness. Fibrosis was quantified in the entire tissue section. Image analysis was performed in Arivis Vision 4D software (Germany). Each image was subjected to a standard threshold to represent whole tissue area and fibrotic tissue area based on SHG signal intensity. Higher SHG signals represented fibrotic areas. Fibrosis was quantified as a percentage of fibrotic volume to total tissue volume.

### Collagen IHC staining

To assess Type I Collagen expression, a chromogenic monoplex IHC assay was developed using a goat anti-collagen type I polyclonal antibody (Southern Biotech, 1310-01; 4 μg/mL, 20 min). Slides were stained using a Ventana Discovery Ultra IHC/ISH research slide staining system with a heated antigen retrieval pretreatment step (Cell Conditioner 1). Signal was detected using OmniMap anti-goat HRP (Ventana #760-4647), and Discovery Detection ChromoMap DAB (Ventana #760-159); and the slides were counterstained with hematoxylin. Stained slides were digitally scanned at ×20 magnification using an Aperio ScanScope AT Turbo brightfield scanner (Leica Biosystems Inc., Buffalo Grove, IL), and the proportion of collagen in each section was quantitated using Halo™ (v2.2.1870, Indica Labs, Corrales, NM) image analysis software.

### Slide preparation

Mouse tissues (lungs, kidneys, and heart) were freshly collected and embedded in OCT. OCT blocks were sectioned at 5 µm on a cryostat. Sections were air-dried at room temperature for 30 min, fixed in acetone for 10 min, and placed in a desiccator overnight. Sections were counterstained with 16 µL DAPI (Invitrogen #D3571) diluted in 200 mL Dako 10X wash buffer (#S3006) for 7 min and then rinsed in running dH_2_O. Glass coverslips were applied to slides with ProLong Gold Antifade Mountant (ThermoFisher Scientific #P36934). Slides were covered and stored in a dark place for at least 24 h prior to confocal analysis.

### Confocal laser microscopy

Immunofluorescence detection by confocal microscopy was used to exam the biodistribution of injected αPV1, a monoclonal antibody directly conjugated with fluorescence fluorophore (Alexa Fluor 680 SAIVI Rapid Antibody Labeling Kit, Life Technologies, Grand Island, NY) in mouse tissues during a mechanism of action study. All slides were evaluated and photographed on a Leica TCS SP5 confocal microscope (Leica Microsystems, Inc., Buffalo Grove, IL) using the ×20 plan objective. The PV1 antibody localization in the tissue is visualized by a fluorescence dye (Alexa 680) with an appropriate excitation filter.

### Histopathologic analysis

At the end of the experiments, the animals were euthanized and the RIGHT/LEFT lobe from each lung were extracted and immediately fixed in 10% neutral buffered formalin. Samples were stained with hematoxylin–eosin and the fibrotic lungs were scored using a modified Ashcroft scale as previously described^26^.

### PV1 immunohistochemistry

FFPE human tissue sections were deparaffinized and treated with 3% hydrogen peroxide in methanol to block endogenous peroxidase and antigens were unmasked with a heat-induced epitope retrieval (HIER) method performed in a Biocare Medical (Concord, CA) pressure cooker containing Dako retrieval solution CB6 (PH6) with temperature at 119 °C for 6.5 min. Dako wash buffer was used for all rinse steps and the samples were stained on a Dako Autostainer. They were blocked with 2% goat serum (Fish Gelatin—Lab standard blocking solution) for 30 min, and the primary antibodies were polyclonal Rabbit anti-PLVAP (Sigma-Aldrich, HPA 002279, SAINT LOUIS MO). The primary antibody was visualized with Dako anti-Rabbit EnVision HRP-labeled polymer (Dako K4003, Lot # 10112992), respectively, and ImmPACT DAB (cat# Sk-4105, Vector Labs Burlingame, CA). All slides were examined by a board-certified pathologist using an upright light microscope (Nikon 80i).

### Antibody quantification

The serum of the animals from the imaging study was collected and the αPV1 and isotype antibodies were quantified. The antibody quantification was done using 96-well ELISA plates (Half Area Clear Flat Bottom Polystyrene High Bind Microplate—Corning) coated with Donkey anti-human Fc antibody (Jackson ImmunoResearch Laboratories, West Grove, PA, USA) 1 μg/mL overnight at 4 oC, plates were then blocked using SuperBlock (Thermo Scientific) and the samples were detected using anti-human Fab (Jackson ImmunoResearch Laboratories, West Grove, PA, USA) 1:10,000. The absorbance at 450 nm was measured using a microtiter plate reader. The resulting data were analyzed using Prism 5 software (GraphPad, San Diego, CA).

### Western blotting

Blood serum was isolated by cardiac puncture and whole tissue homogenates were isolated from heart, kidney, liver, and lung. Proteins (50 μg per lane) were separated by SDS–PAGE under reducing conditions and transferred onto nitrocellulose membranes. Recombinant PV1 (25 ng) was used as a positive control. Membranes were blocked with 1% casein blocking solution (G-Bioscience) and probed with αPV1 (1 μg/mL) diluted in blocking buffer for 1 hour at room temperature followed by a mouse anti-human Fc/HRP-conjugate (Jackson ImmunoResearch Laboratories, West Grove, PA, USA) for 1 h at room temperature (1:10,000). Proteins were visualized using SuperSignal ELISA Pico chemiluminescent substrate according to the manufacturer’s instructions (Thermo Fisher, USA). Uncropped western blots are shown in Supplementary Figure 6.

### Gene expression

Mouse tissues (up to 100 mg) were frozen and homogenized with 1 mL of TRIzol (Invitrogen) using a TissueLyser II (Qiagen, Valencia, CA, USA) according to the manufacturer’s instructions. After the chloroform extraction, the RNA was isolated using an RNeasy kit (Qiagen, Valencia, CA, USA) according to manufacturer’s protocol. First-strand cDNA was synthesized with SuperScript III First-Strand Synthesis SuperMix with up to 5 μg of total cellular RNA and 50 ng of random hexamers (Invitrogen, USA). After synthesis, the reaction mixture was immediately subjected to quantitative polymerase chain reaction (qPCR) using TaqMan primers and TaqMan Fast Advanced Master Mix using the QuantStudio™ 6 Flex Real-Time PCR System (Applied Biosystem). Fold change of the gene expression was measured comparing the expression of tested genes with that of housekeeping genes (β-actin and GAPDH) and expressed as fold change in gene expression as 2^−ΔΔCt^ values (ΔΔCt = ΔCt_treated_−ΔCt_control_) with the highest PV1 expressing tissue in the panel normalized to 1. For the detection of human PV1 gene expression, the Human Multiple Tissue cDNA Panel I (Clontech) was used.

Bleomycin-treated mice having relative mRNA levels of fibrotic genes (Supplementary Figure 5) that were consistent outliers (as determined by Dixon’s test) were excluded from IHC, Collagen, and SHG data sets. One mouse in the isotype group, one mouse in the αPV1 group and one mouse in the αPV1-PGE2 group were excluded because of these criteria.

### Antibody-PGE2 labeling

Purified αPV1 antibody was chemically linked to PGE_2_ using zero-length cross-linker 1-ethyl-3-[3-dimethylamino-propyl] carbodiimide hydrochloride (EDC) according to the manufacturer’s instructions. A non-target isotype control, was also inked to PGE_2_ and injected into a control group of mice. In brief, the antibodies (10 mg) were incubated with PGE_2_ (2 mg) and EDC (1 mg) in MES pH4.7 for 2 h at room temperature. Unconjugated PGE_2_ and EDC were removed using Zeba spin desalting columns, 7K MWCO (Thermo Scientific). The amount of PGE2 linked to the antibody was calculated using a PGE2 ELISA Kit (Cayman Chemical) and the activity of the PGE_2_ attached to the antibody was evaluated using EP4 Receptor (rat) Reporter Assay Kit (Cayman Chemical) according to the manufacturer’s instructions (using HEK293 cells).

### Bleomycin model of lung fibrosis

Lung fibrosis was induced by a 100 mg/kg dose of bleomycin delivered via osmotic pump (Alzet) at a rate of 1 μl/h for 1 week that was surgically inserted under the back skin of C57BL/6 mice (Harlan) on day 0. Bleomycin-induced lung injury and inflammation causes progressive lung remodeling and fibrosis that typically peaks at week 4. Mice (*n* = 8 per group) were treated with 6 mg/kg of antibody starting at one day before bleomycin initiation. A sample size of at least 7 in each group will have 80% power to significantly detect a 10% improvement in lung function and collagen content, knowing that a 10% improvement in lung function after IPF therapy is considered a favorable response by the American Thoracic Society. At week 4, blood and lungs were collected from each mouse. One lobe of lung was frozen for histopathological analysis and rest of the lung was used for analyzing fibrosis biomarkers (protein and mRNA analysis). Both hematoxylin and eosin (H&E) and Masson’s trichrome were used to evaluate extent of collagen deposition in lungs. Histological evaluations were scored by a modified Ashcroft scale system validated in a bleomycin-induced lung fibrosis^26^. One animal in the αPV1-PGE_2_ group died before completion of the study.

### SPR measurements

Antibody affinity to mouse PV1 was determined by SPR with a ProteOn^TM^ XPR36 (Bio-Rad, Hercules, CA, USA). Mouse PV1 was coupled to a GLC sensor chip using a ProteOn^TM^ amine coupling kit (Bio-Rad) according to the manufacturer’s instructions. Excess reactive groups were blocked with a 2-min injection of 1 M ethanolamine. PV1 was immobilized at a surface density of ~200 resonance units. Antibodies were injected at a flow rate of 100 μl/min. One channel was always left unmodified to provide a blank reference surface. Dilutions and binding experiments were carried out at 25 °C in phosphate-buffered saline (PBS pH 7.2). Dissociation constants (*K*_D_s) were determined by fitting the kinetics for association and dissociation employing a 1:1 Langmuir model.

## Supplementary information

Description of Supplementary Data

Supplementary Data 1

Reporting Summary

Supplementary Figures

## Data Availability

The authors declare that all data supporting the findings of this study are available within the paper and its supplementary information. Source data underlying the graphs and charts presented in the main figures is available in Supplementary Data 1.
