# Supplementary material for: Targeted drug delivery via caveolae-associated protein PV1 improves lung fibrosis
Source: Commun Biol. 2019 Mar 7;2:92. doi: 10.1038/s42003-019-0337-2 (PMC6405929; doi:10.1038/s42003-019-0337-2)
Supplement: Supplementary file 1 — Description of Supplementary Data [file 42003_2019_337_MOESM1_ESM.pdf]

Descriptions of Additional Supplementary File for Marchetti *et al.*

File Name: Supplementary Data 1

Description: The source data underlying the graphs and charts presented in main figures. The following source data are captured: relative mRNA expression, *ex vivo* imaging (tissue florescence relative to muscle or average radiant efficiency), antibody quantitation from serum, Ashcroft scores of H&E stained lung images, percent of lung with Second Harmonics Generated signal, Collagen positive lung area.   These data underlie the graphs presented in Figures 1-6.
