# Supplementary material for: Targeted drug delivery via caveolae-associated protein PV1 improves lung fibrosis
Source: Commun Biol. 2019 Mar 7;2:92. doi: 10.1038/s42003-019-0337-2 (PMC6405929; doi:10.1038/s42003-019-0337-2)
Supplement: Supplementary file 3 — Reporting Summary [file 42003_2019_337_MOESM3_ESM.pdf]

natureresearch

Corresponding author(s): Dr. M. Jack Borrok

Reporting Summary

Nature Research wishes to improve the reproducibility of the work that we publish. This form provides structure for consistency and transparency in reporting. For further information on Nature Research policies, see Authors & Referees and the Editorial Policy Checklist.

Statistical parameters

When statistical analyses are reported, confirm that the following items are present in the relevant location (e.g. figure legend, table legend, main text, or Methods section).

n/a	Confirmed	
☐	☒	The exact sample size (*n*) for each experimental group/condition, given as a discrete number and unit of measurement
☐	☒	An indication of whether measurements were taken from distinct samples or whether the same sample was measured repeatedly
☐	☒	The statistical test(s) used AND whether they are one- or two-sided<br>*Only common tests should be described solely by name; describe more complex techniques in the Methods section.*
☒	☐	A description of all covariates tested
☐	☒	A description of any assumptions or corrections, such as tests of normality and adjustment for multiple comparisons
☐	☒	A full description of the statistics including central tendency (e.g. means) or other basic estimates (e.g. regression coefficient) AND variation (e.g. standard deviation) or associated estimates of uncertainty (e.g. confidence intervals)
☐	☒	For null hypothesis testing, the test statistic (e.g. *F*, *t*, *r*) with confidence intervals, effect sizes, degrees of freedom and *P* value noted<br>*Give P values as exact values whenever suitable.*
☒	☐	For Bayesian analysis, information on the choice of priors and Markov chain Monte Carlo settings
☒	☐	For hierarchical and complex designs, identification of the appropriate level for tests and full reporting of outcomes
☒	☐	Estimates of effect sizes (e.g. Cohen's *d*, Pearson's *r*), indicating how they were calculated
☐	☒	Clearly defined error bars<br>*State explicitly what error bars represent (e.g. SD, SE, CI)*

Our web collection on statistics for biologists may be useful.

Software and code

Policy information about availability of computer code

Data collection	Percent collagen in tissue sections was quantified using Halo™ (v2.2.1870, Indica Labs, Corrales, NM) image analysis software.
Data analysis	Statistical analysis was performed using GraphPad Prism (v.5 or v.6)

For manuscripts utilizing custom algorithms or software that are central to the research but not yet described in published literature, software must be made available to editors/reviewers upon request. We strongly encourage code deposition in a community repository (e.g. GitHub). See the Nature Research guidelines for submitting code & software for further information.

Data

Policy information about availability of data

All manuscripts must include a data availability statement. This statement should provide the following information, where applicable:

- Accession codes, unique identifiers, or web links for publicly available datasets
- A list of figures that have associated raw data
- A description of any restrictions on data availability

We have included the following data availability statement: "The authors declare that all data supporting the findings of this study are available within the paper and its supplementary information." Additionally, we have made the data comprising figures available in supplementary information.

Field-specific reporting

Please select the best fit for your research. If you are not sure, read the appropriate sections before making your selection.

☒ Life sciences ☐ Behavioural & social sciences

For a reference copy of the document with all sections, see nature.com/authors/policies/ReportingSummary-flat.pdf

Life sciences

Study design

All studies must disclose on these points even when the disclosure is negative.

Sample size	Power calculation is a prerequisite for any animal experiment according to the IACUC regulations. A sample size of 7 in each group will have 80% power to significantly detect a 10% improvement in lung function and collagen content, knowing that a 10% improvement in lung function after IPF therapy is considered a favorable response by the American Thoracic Society. This number is calculated from in-house data looking at change in hydroxyproline levels in lung where fibrosis is induced by bleomycin-containing osmotic mini-pump.<br><br>Briefly, a sample size of 7 in each group will have 80% power to detect a difference in means of 28.918 (the difference between a Group 1 mean, µ1, of 192.790 and a Group 2 mean, µ2, of 163.872) assuming that the common standard deviation is 19.090 using a two group Satterthwaite t-test with a 0.050 one-sided significance level.
Data exclusions	Outliers were determined by Dixon's test. Bleomycin treated mice having relative mRNA levels of fibrotic genes (Supplementary Figure 5) that were consistent outliers (as determined by Dixon's test) were excluded from IHC, collagen and SHG data sets. One mouse in the isotype group, one mouse in the aPV1 group and one mouse in the aPV1-PGE2 group were excluded because of this criteria. Additionally, for the SHG data generation, two samples in the isotype group were excluded due to power fluctuations noted during the 12 hours multi-photon laser scan.
Replication	All findings shown have been reproduced in at least two independent experiments. Individual n values are shown in each figure.
Randomization	Animals were randomly assigned to the respective body weight matched groups.
Blinding	Investigators were blinded for data analysis & histological scoring.

Materials & experimental systems

Policy information about availability of materials

n/a	Involved in the study
☐	☒ Unique materials
☐	☒ Antibodies
☐	☒ Eukaryotic cell lines
☐	☒ Research animals
☒	☐ Human research participants

Unique materials

Obtaining unique materials	All unique materials can be readily generated from commercial sources. Antibody sequences are given or are commercially available

Antibodies

Antibodies used	Meca32, bxcell, Cat # BE0200
Validation	Validation was done in-house. Both commercial and in house generated Meca32 constructs bound to murine PV1, but not the human PV1 (as expected).

Eukaryotic cell lines

Policy information about cell lines

Cell line source(s)	HEK293 (ATCC) cells were used for PGE2 activity level testing in EP4 Receptor (rat) Reporter Assay Kit (Cayman Chemical). HEK293F cells (Invitrogen) were used for antibody and antigen production.

Authentication	HEK293 cell lines were authenticated by the vendor and banked on site.
Mycoplasma contamination	HEK293 cells are periodically tested for mycoplasma contamination.
Commonly misidentified lines (See ICLAC register)	HEK derived cells are on the list of commonly misidentified cell lines. In this manuscript, HEK cells were used for a confirmatory reporter gene assay and for antibody production.

Research animals

Policy information about studies involving animals; ARRIVE guidelines recommended for reporting animal research

Animals/animal-derived materials	All animal studies were approved by the MedImmune IACUC and were conducted in an Association for Assessment and Accreditation of Laboratory Animal Care–accredited facility in compliance with US regulations governing the housing and use of animals. C57Bl6 female mice from Harlan, age: 8-14 weeks.

Method-specific reporting

n/a	Involved in the study
☒	☐ ChIP-seq
☒	☐ Flow cytometry
☒	☐ Magnetic resonance imaging
