# Supplementary material for: Targeted drug delivery via caveolae-associated protein PV1 improves lung fibrosis
Source: Commun Biol. 2019 Mar 7;2:92. doi: 10.1038/s42003-019-0337-2 (PMC6405929; doi:10.1038/s42003-019-0337-2)
Supplement: Supplementary file 4 — Supplementary Figures [file 42003_2019_337_MOESM4_ESM.pdf]

Supplementary Figure 1

Untreated Bleomycin Treated

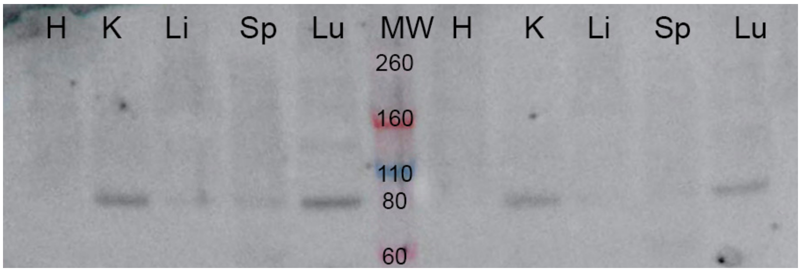

Figure S1 Western blot analysis of tissues from untreated and bleomycin treated mice. Homogenized tissue lysate was generated from naïve mice or mice that had been treated with bleomycin for 28 days administered via osmotic pump. 50ug of total protein from tissue lysates was loaded in each lane. PV-1 dimer was detected in lungs and kidneys via Meca32 antibody. H = Heart, K = Kidney, Li = Liver, Sp = Spleen, Lu = Lung.

Supplementary Figure 2

A

VK

DIQMTQTPSSMSASLGERVTLSCGTSQGVNLFLNWYQQKPDGTIKPLIFFTSHLQSGVPSRFSGSGSGTAYSLTISSLEPEDFAVYYCQQYDSSPPTFGGGTKLYLK

VH

EVQLVESGGGLVQPGRSMKLSCAASGFTFSDYYMAWVRQAPKKGLEWVASISYEGNKTYYGDSVKGRFTISRDNAKSILYLQMNSLKSEDTATYYCARQSYSSYLFDYWGQGVM

B

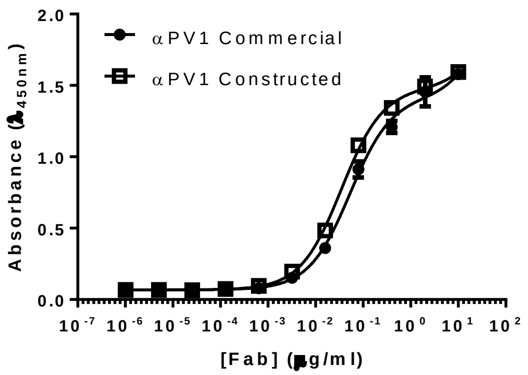

C

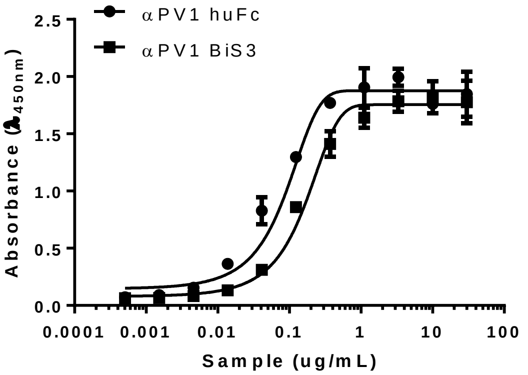

D

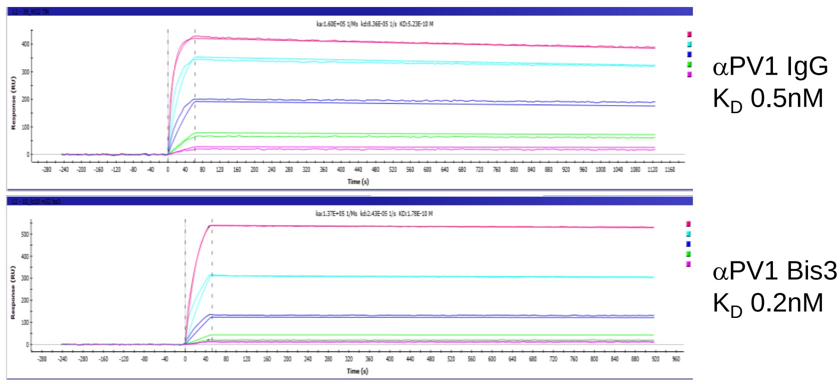

Figure S2 ***De novo*** **sequencing of Meca32.** Sequence of αPV1 (Meca32) variant 1 (A) with CDRs (underlined) was determined by *de novo* mass spec sequencing. ELISA binding of variant 1 Fab to immobilized mouse PV-1 was determined to be similar to that of commercial Meca32 Fab (B). Binding of bispecific Meca32 (Bis3 format) was also assessed by PV-1 ELISA compared to an IgG version of Meca32 variant 1 with a human Fc (C). K_D values for both the αPV1 IgG and the Bis3 bispecific αPV1 were determined using surface plasmon resonance (SPR). Both K_D values were similar (0.2nM for αPV1 Bis3 and 0.5nM for αPV1 IgG).

Supplementary Figure 3

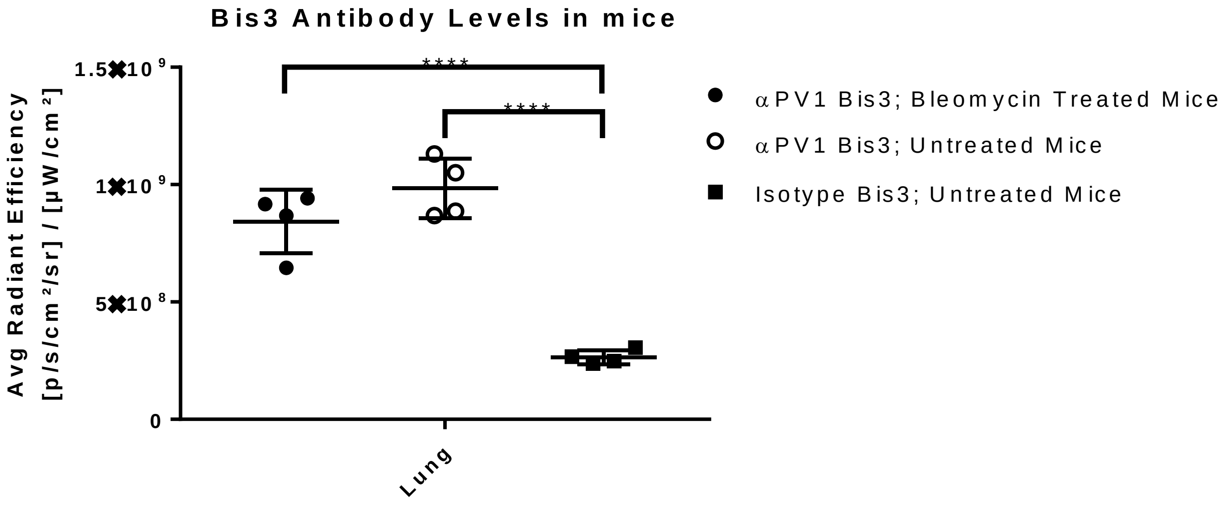

Figure S3 PV1 mediated accumulation in Bleomycin-treated and untreated mice. C57BL/6 mice were implanted with osmotic pumps delivering bleomycin for 28 days. These mice, as well as untreated C57BL/6 were then dosed with Alexa 750 labeled αPV1 Bis3 antibodies. After 24 hours, *ex vivo* fluorescence of lungs was measured. No significant difference was observed between αPV1Bis3 treated groups indicating bleomycin treatment does not significantly affect lung accumulation. However, both groups had significantly higher accumulation than an isotype Bis3 (****$p < 0.0001$; n=4).

Supplementary Figure 4

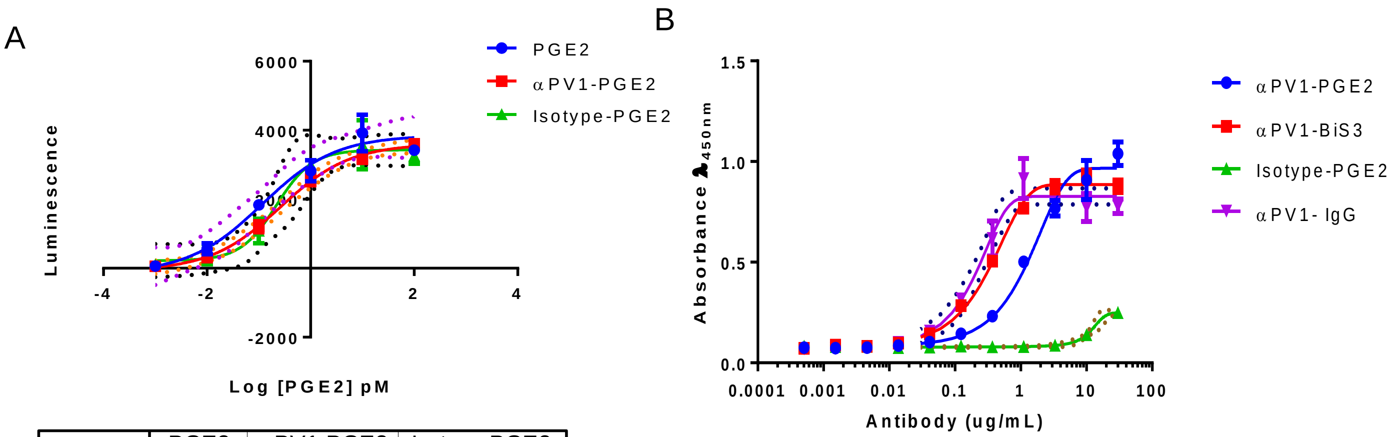

	PGE2	αPV1-PGE2	Isotype-PGE2
IC50	0.1159	0.2599	0.2188

Figure S4 PGE2 activity and PV1 binding of the antibody conjugates. Both PV1 and isotype antibody-PGE2 conjugates retain the PGE2 activity (A). After the antibody conjugation, Anti-PV1-PGE_2 is still able to activate EP4 Receptor, with similar EC50 than the isotype-PGE_2. PV1 binding (B) was observed for αPV1 mAb, bispecific antibody and PGE2 construct but isotype-PGE2 did not show binding to PV1.

Supplementary Figure 5

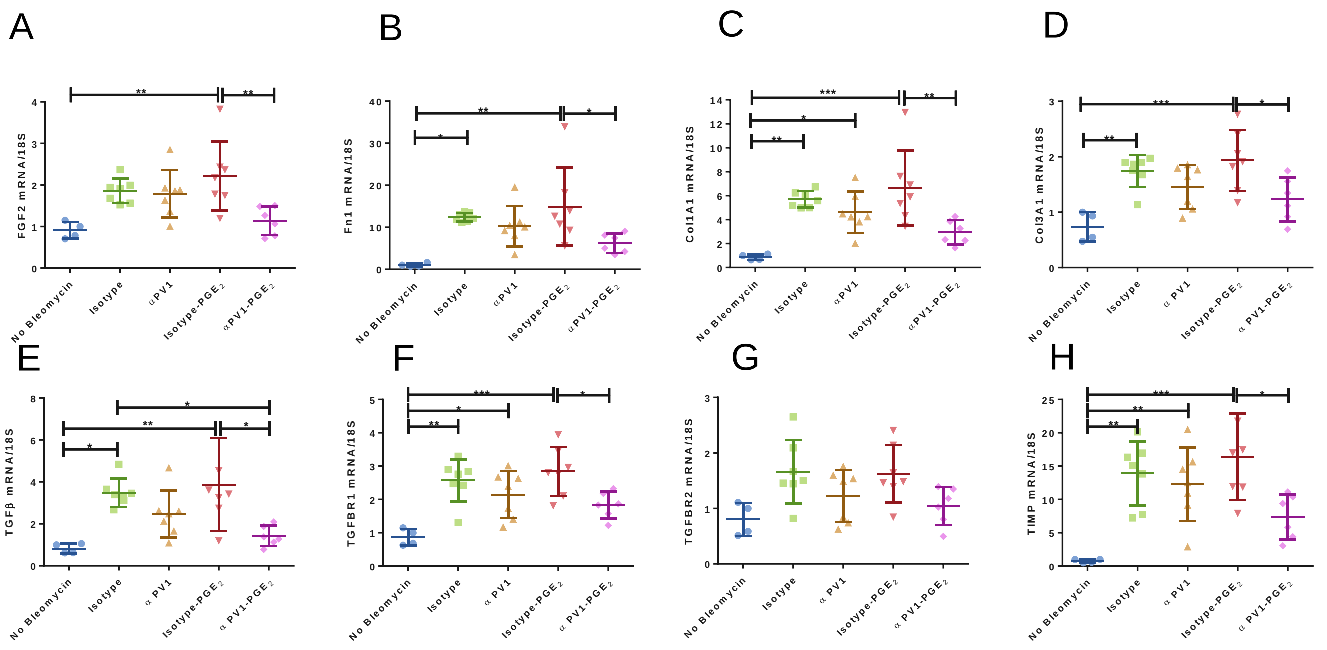

Figure S5 Gene expression of fibrosis markers, measured by the quantitative polymerase chain reaction (qPCR). Means + SD are shown. In bleomycin induced lung fibrosis, αPV1-PGE2 is able to inhibit fibroblast proliferation, here shown by reducing fibroblast growth factor 2 (FGF2) (A) and extracellular matrix protein synthesis (B) fibronectin (Fn1) and (C and D) collagens (Col1A1 and Col3A1). PGE2 also demonstrate improvement in the transforming growth factor (TGF)-mediated cell signaling. (E) TGF-β and it’s receptors (F) TGFBR1 and (G) TGFBR2. The fibrogenic factor TIMP1 (H) is also reduced by αPV1-PGE2. One Way ANOVA (Tukey test) was used to evaluate the statistical significance. *$p < 0.05$, **$p < 0.01$ and ***$p < 0.001$; n=8

Supplementary Figure 6

A

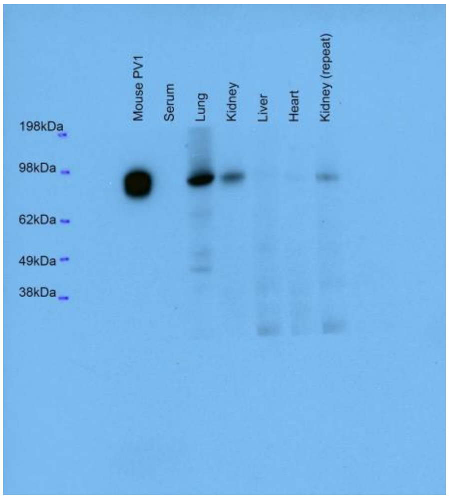

B

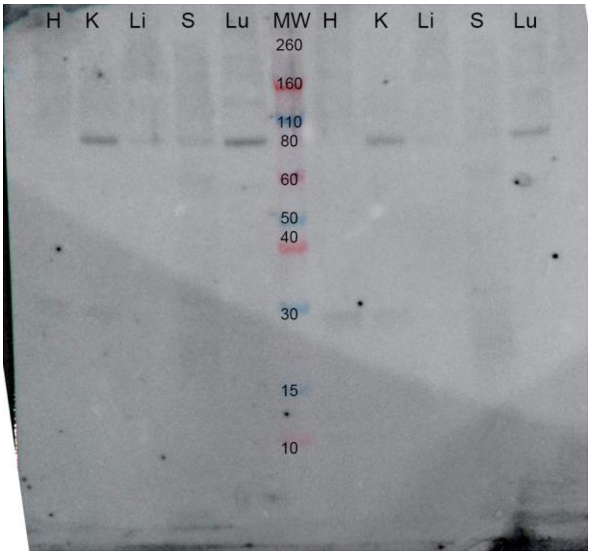

Figure S6 Uncropped Western Blot images. Uncropped Blot for Figure 1B (A) and Supplementary Figure 1 (B) are shown.
